# The biological functions of ginsenoside and its applications in animal husbandry

**DOI:** 10.3389/fvets.2025.1648629

**Published:** 2025-09-05

**Authors:** Yongqiang Li, Guohui Zhang, Jingwei Wang, Jiahui Li, Yuhe Liu, Shengsheng Pan, Shihui Chang, Yang Gao

**Affiliations:** ^1^College of Life Science, Baicheng Normal University, Baicheng, China; ^2^Changchun Borui Science & Technology Co., Ltd, Changchun, China; ^3^College of Animal Science and Technology, Jilin Agricultural University, Changchun, China

**Keywords:** ginsenoside, biological function, animal production, feed additives, application

## Abstract

Ginsenoside, as the pivotal bio-active constituents derived from ginseng, exhibit multifunctional biological properties including antioxidant, anti-inflammatory, immune regulation and stress-alleviating effects. Ginsenosides modulate immune responses, enhance metabolic regulation, and exert antioxidant effects through multiple pathways, improving animal health, meat quality and productivity. The purpose of this article is to provide solutions for the development of new feed additives under the premise of a complete ban on the use of antibiotics. Consequently, ginsenosides represent a premium botanical resource for feed additive applications in modern livestock and poultry production. This paper reviews the structural classification, source, biological function and application of ginsenoside in animals, in order to provide a reference for the rational use of ginsenoside in animal husbandry.

## 1 Introduction

Ginseng, discovered in China more than 5,000 years ago, is a perennial herb which is called the “king of the herb” (1, 2). In China, Changbai Mountain in Jilin Province is the area where the natural growth yields is the highest (3). In recent years, with the continuous optimization of the extraction and separation technology of Chinese herbal medicine, ginsenoside, the main medicinal active ingredient in ginseng, has also attracted much attention from researchers. Ginsenosides are a kind of natural steroid glycosides and triterpenoid saponins, which are often used as markers to determine the medicinal value of ginseng (4, 5). Ginsenosides have multiple biologically active functions, including immune regulation (6), protection of the central nervous system and cardiovascular health (7, 8), anti-inflammatory (9), antioxidant (10) and even anti-cancer (11), but suffer from drawbacks including poor water solubility, short half-life, and low bioavailability. Currently, the specific mechanisms of action for many ginsenosides remain were unclear, and many researchers are also working in this area to solve these problems. At present, relevant research reports have been published on the application of ginsenosides in animal production (12). Based on the research reports, this article summarizes the chemical structure, classification and sources, biological functions, and applications of ginsenosides in animal production, in order to develop a new natural green feed additive in utilizing animal husbandry.

## 2 Chemical structure and classification of ginsenosides

**Figure 1:**
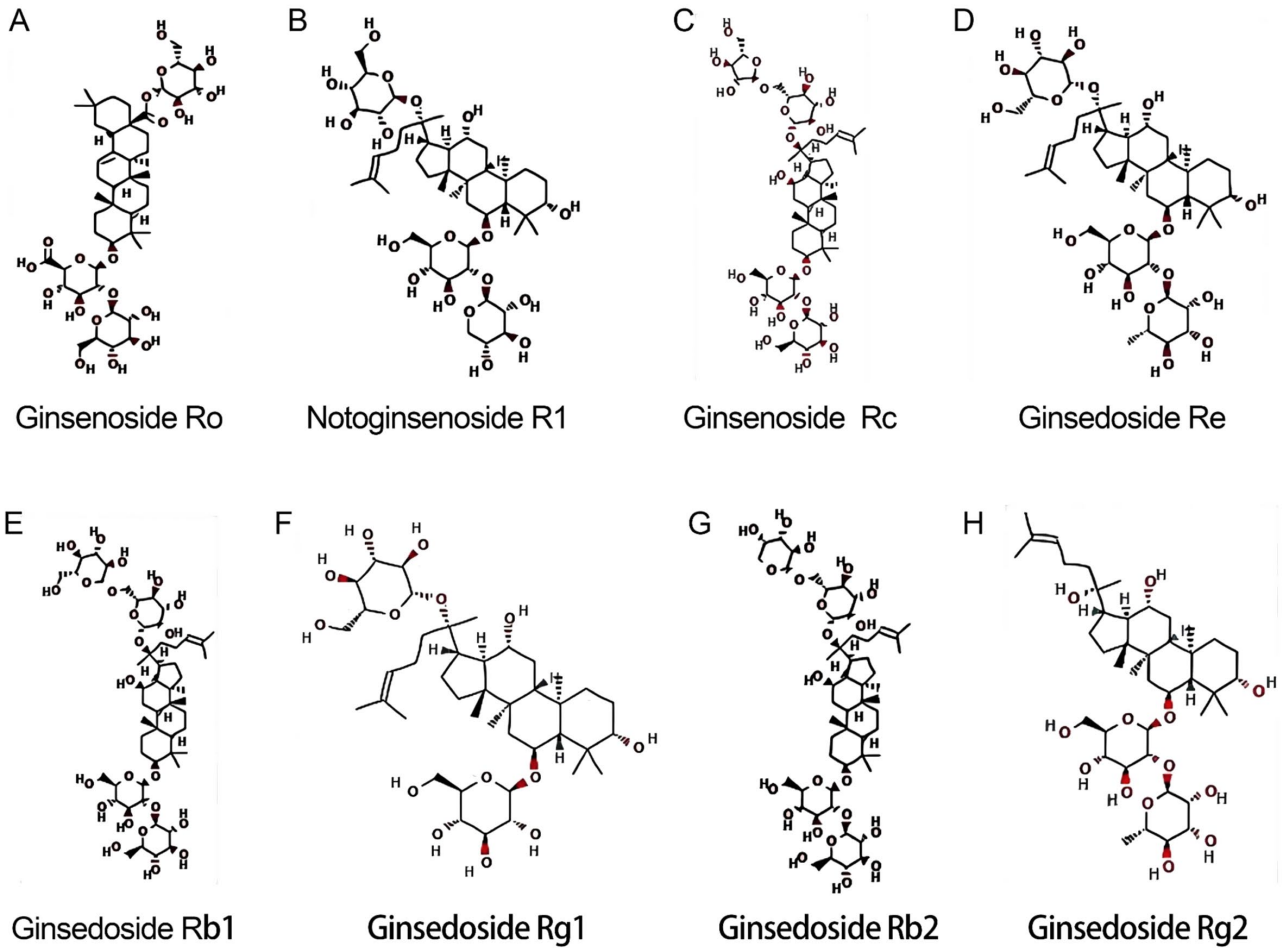
Molecular structures of 8 common ginsenosides. **(A)** Ginsenoside Ro. **(B)** Notoginsenoside R1. **(C)** Ginsenoside Rc. **(D)** Ginsenoside Re. **(E)** Ginsenoside Rb1. **(F)** Ginsenoside Rg1. **(G)** Ginsenoside Rb2. **(H)** Ginsenoside Rg2.

The basic structure of ginsenosides is similar, consisting of a 17-carbon-atomic paeonol steroid nucleus, the structure of which was first discovered by a Japanese researcher in the 1960s (1). According to their mobility on thin layer chromatography plates, they can be divided into four major categories: 20(S)-Protopanaxadiol (PPD), 20(S)-Protopanaxatriol (PPT), C17 Side-chain Varied (C17SCV) and oleanolic acid (OA). PPD, PPT and C17SCV are the main types of ginsenosides, and their compositions vary significantly in different parts of ginseng (12). Sun et al. (13) identified a total of 408 ginsenosides by ultra-high performance liquid chromatography-time-of-flight mass spectrometry (UPLC-TOF-MS) qualitative analysis, of which 8 common saponins were found in all parts of the whole ginseng plant (12), as shown in Figure 1, including 3 types of PPD (ginsenoside Rb1, ginsenoside Rb2, ginsenoside Rc), 4 types of PPT (ginsenoside Re, ginsenoside Rg1, ginsenoside Rg2, Notoginsenoside R1), and 1 oleanolic acid type (ginsenoside Ro).

## 3 The biological functions of ginsenosides

Ginsenosides exert multifaceted regulatory effects on animals primarily through modulation of critical signaling pathways and interaction with cellular receptors. Ginsenosides can activate Nrf2 signal pathway, promoting its nuclear translocation and binding to the Antioxidant Response Element (ARE), leading to up-regulated expression of antioxidant enzymes (HO-1, SOD, CAT, GSH-Px) (14). Ginsenosides can also suppress pro-inflammatory cytokines (TNF-*α*, IL-1β, IL-6) by inhibiting IκBα degradation and nuclear translocation of NF-κB (15, 16). As well as modulate c-jun N-terminal kinase (JNK) (17), ERK (18), and p38 signaling pathways (19), reducing inflammation and cellular stress responses. In the part of anti-stress, ginsenosides can regulate unfolded protein response (UPR) sensors and reducing excessive ER stress-induced apoptosis (20). The main biological functions of ginsenosides was shown as followed.

### 3.1 Antioxidant

Reactive Oxygen Species (ROS) are highly reactive molecules containing oxygen, generated through both normal cellular metabolism and exposure to external factors. This complex interplay of metabolic byproducts, enzymatic reactions, conversions, and environmental exposures constitutes the major pathways of ROS formation within biological systems (21, 22). They are unstable compounds that mainly originate from the oxidative phosphorylation process in the mitochondrial electron transport chain. They are usually produced as a byproduct of cellular metabolism and always play an important role in signal transduction. However, their excessive production can cause cellular oxidative damage (23). Under the condition of intensive farming process in recent years, animals are easily affected by external factors such as weaning, vaccination and temperature, which cause ROS to accumulate *in vivo*, damaging macro-molecules such as lipids and proteins (24, 25), leading to an imbalance of oxidative and antioxidant homeostasis, finally ultimately causing oxidative stress (26). A study has found that ginsenosides can relieve oxidative stress by scavenging free radicals, inhibiting the production of nitric oxide (NO), inducing catalase (CAT) and superoxide dismutase (SOD) gene expression and reducing lipid peroxidation (27). Similarly, ginsenoside Rb1 can exert antioxidant effects in ischemic hippocampal neurons by increasing endogenous antioxidant enzymes, thereby protecting the central nervous system (28). Anti-NF-E2-related factor 2 (Nrf2) plays a key role in regulating the expression of antioxidant-related genes (29). Under normal physiological conditions, Nrf2 usually exists in the cytoplasm together with Kelch-like epichlorohydrin-associated protein 1 (Keap1). When animals are under oxidative stress, Nrf2 binds to the antioxidant response element (ARE) to initiate the transcription of antioxidant enzyme genes, up-regulating the expression of genes encoding the second type of enzymes and antioxidant proteins, including NAD (P)H: quinone oxidoreductase 1 (NQO1), glutamate-cysteine ligase catalytic subunit (GCLc) and heme oxygenase-1 (HO-1), further enhancing the antioxidant capacity of cells escaping from oxidative damage (30). A study by Liu et al. (31) showed that 25 μM ginsenosides can significantly activate the Nrf2/HO-1 antioxidant pathway, enhance the activity of various antioxidant enzymes such as GSH-Px and SOD, and effectively alleviate cellular oxidative stress damage. The above studies showed that ginsenosides can exert antioxidant effects by activating the Nrf2 antioxidant pathway *in vivo* and up-regulating the gene expression of various antioxidant enzymes such as SOD, CAT, HO-1, NQO1 and GCLc.

### 3.2 Anti-inflammatory

Inflammation is an immune response to infection in animals. When animals are infected by pathogens, cell surface receptors (such as TLRs) activate the nuclear factor κB (NF-κB) and activator protein 1 (AP-1) signaling pathways, releasing pro-inflammatory cytokines and inducing inflammatory responses (32, 33). Studies have shown that ginsenoside Rb1 can inhibit the Toll-like receptor 4 (TLR4) pathway and protect mice from LPS induced liver damage (34), it alleviated hypoxia-induced cardiomyocyte apoptosis and inflammatory response in rats (35). Ginsenosides significantly inhibits LPS-induced NO release in RAW264.7 macrophages in a dose-dependent manner (0–100 μmol/L), achieving near-complete suppression at 100 μmol/L, while downregulating mRNA expression of pro-inflammatory mediators (36). Similarly, 20 mg/kg ginsenoside reduces chronic inflammatory pain in mice by suppressing TLR4/NF-κB signaling, decreasing spinal expression of IL-1β, TLR4, and NF-κB by 40–50%, while elevating mechanical pain thresholds by 2.5-fold and prolonging rotarod endurance by 80% (37). Ginsenoside Rk1 can also inhibit NF-κB and Janus kinase 2 (Jak2) signal transduction, meanwhile inhibit the production of NO, tumor necrosis factor (TNF-*α*), interleukin-6 (IL-6), MCP-1, and interleukin-1β (IL-1β) induced by LPS in the mouse mononuclear macrophage cell line RAW264.7, thereby alleviating the inflammatory response (38). NF-κB is a transcription factor associated with inflammatory response (30). It interacts with the Nrf2 pathway. Nrf2 up-regulation can inhibit NF-κB activation, at the same time NF-κB mediated transcription can also inhibit Nrf2 activation and reduce antioxidant capacity (39). As an exogenous regulatory factor, ginsenosides can activate the Nrf2 antioxidant defense system through the PI3K/Akt pathway and inhibit the NF-κB inflammatory signaling pathway, thereby alleviating LPS induced blood–brain barrier (BBB) damage (40). Ginsenoside Ro can increase the expression of HO-1 in macrophages, activate the Nrf2 signaling pathway, and reduce the expression of LPS induced cyclooxygenase-2 (COX-2), thereby improving the antioxidant capacity (41). In summary, ginsenosides can exert anti-inflammatory effects by activating the antioxidant defense system to inhibit inflammatory responses or directly acting on inflammatory signaling pathways.

### 3.3 Immune regulation

Ginsenosides play an important role in enhancing humoral immunity and cellular immunity. Immunoglobulin is the main mediator of humoral immunity. Immunoglobulin is the main mediator of humoral immunity. Ginsenosides can promote the production of serum immunoglobulin G (IgG) and immunoglobulin M (IgM) in mice. When the feeding dose was 60 mg/kg, 120 mg/kg and 240 mg/kg, the serum IgG content was significantly increased by 23.39, 24.29 and 26.39%, respectively, compared with the control group, and the IgM content was increased by 32.47, 33.17 and 38.33%, respectively, compared with the control group (42). In addition, ginsenosides are also widely used in vaccine adjuvants. Oral administration of ginseng stem and leaf saponins (GSLS) can significantly enhance the immune efficacy of infectious bursal disease virus (IBDV) vaccines and Newcastle disease virus (NDV) vaccines in chickens (43). A study by Su et al. (44) showed that ginsenoside Re, as a vaccine adjuvant, can enhance the immune response of mice to inactivated rabies vaccine (RV) by enhancing cellular and humoral immune responses, thereby increasing the serum antibody level after vaccination. Yuan et al. (45) found that ginsenoside Rg1 had adjuvant properties in stimulating IgG, splenocyte proliferation, and mRNA expression of cytokines IFN-*γ* and IL-4, as well as the expression of cell surface marker TLR4 in the HBsAg-immunized mice. Therefore, ginsenosides can improve immune function by increasing serum immunoglobulin levels and related cytokine production.

### 3.4 Anti-stress

**Figure 2:**
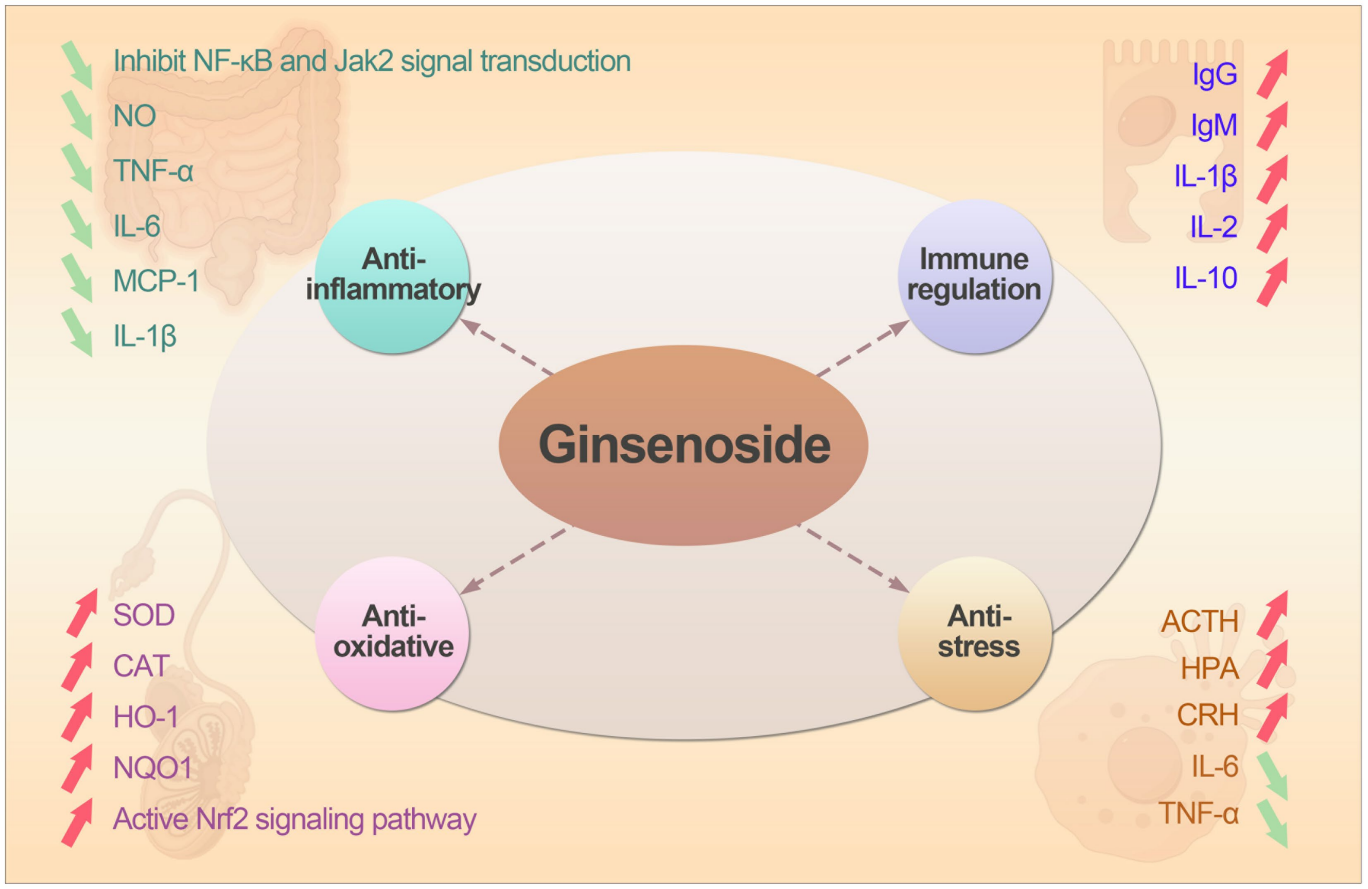
The the biological functions and mechanism action of ginsenosides.

In large-scale farming environments, stress often occurs in early weaning piglets, heat stress in dairy cows and immune stress in broiler chicken. These stress from the external environment can cause an increase in ROS produced by the mitochondrial respiratory chain in animals, leading to imbalance between the oxidative and antioxidant systems (46). Therefore, whether weaning stress or heat stress, it is ultimately cellular oxidative stress. Li et al. (47) showed that ginsenoside Rg1 can protect H9c2 cells from Hypoxia/Re-oxygenation induced apoptosis by alleviating oxidative stress injury, which depended largely on subsequent Nrf2 nuclear translocation and up-regulation of HO-1. Similarly, ginsenoside Rb1 can also reduce oxidative stress and cell apoptosis caused by *Staphylococcus aureus* by activating the Nrf2 signaling pathway and inhibiting the mitochondria-mediated apoptosis pathway (48). Immune stress can also have adverse effects on the health of livestock and poultry (49), the mechanism is that immune stress induced by adrenocorticotropic hormone (ACTH) increased directly. When animals are under stress, the hypothalamic–pituitary–adrenal axis (HPA) is activated, the hypothalamus can secrete corticotropin-releasing hormone (CRH) and ACTH (50). A study has shown that ginsenoside Rg3 can reduce serum ACTH levels in broiler chickens under immune stress (51). The mechanism of action may be that ginsenoside Rg3 acts on the HPA axis, stimulating the hypothalamic thermoregulatory center to lower the body temperature of broilers, shorten the fever period, and inhibit the release of pro-inflammatory factors such as IL-6 and TNF-*α* (39), thereby alleviating the immune stress caused by LPS. The biological functions of ginsenosides was shown in Figure 2.

## 4 The application of ginsenosides in animal production

Ginsenosides, primarily administered as feed additives or oral supplements, demonstrate species-specific benefits across livestock: in poultry, doses of 15 mg/kg body weight enhance mucosal immunity, boost antioxidant capacity and improve vaccine efficacy against pathogens (52). In aquatic species such as large yellow croaker, inclusion at 500 mg/kg feed reduces winter mortality by 80%, accelerates post-cold weight gain, and strengthens disease resilience (53). While ruminant applications remain emerging, early evidence suggests potential for metabolic modulation and stress mitigation (54). Conversely, swine research is sparse but proposes roles in gut microbiota optimization and inflammation control (55). Critically, core effects such as immune regulation, antioxidant, anti-inflammatory, underpin these benefits across all species, though ruminant and swine models urgently require targeted validation. The summary of ginsenosides were used in animals was shown in Table 1.

**Table 1:** The summary of ginsenosides were used in animal production.

Species	Dosage	Effects
Swine	10–100 mg/kg	Gut microbiota modulation (SCFA producers like Blautia for enteric virus defense↑) Increase the reproductive performance
Poultry	15–300 mg/kg BW orally 6 mg/kg in water (for ducks)	Enhanced mucosal immunity (↑ sIgA, IELs) Improved antioxidant capacity (↑ SOD, CAT, GSH-Px; ↓ MDA) Reduced oxidative stress induced by cyclophosphamide Growth performance
Ruminants	2.5–10 mg/kg BW	Potential immunomodulation and stress resilience metabolic benefits Milk quality; Growth performance
Aquatic	500 g/ton feed 4 g/kg	Increase growth performance

### 4.1 The application of ginsenosides in swine industry

Ginsenosides, mainly administered as oral supplements or feed additives, demonstrate emerging potential in swine production through multifaceted biological actions, they enhance gut health by modulating microbiota composition (56), strengthen intestinal barrier integrity via up-regulation of tight junction proteins such as ZO-1 and occludin (57), and boost systemic and mucosal immunity by elevating serum IgG/IgA levels and activating TLR4/NF-κB pathways (58), thereby improving vaccine efficacy against pathogens like PRRSV (59) and PEDV (60). Concurrently, they mitigate oxidative stress by elevating antioxidant enzymes (SOD, GSH-Px) via Nrf2 activation while suppressing pro-inflammatory cytokines and preliminary evidence suggests roles in improving growth performance by optimizing nutrient metabolism (61). The intestinal microbiota of early-weaned piglets is easily disturbed, resulting in reduce the abilities of digestion and absorption. Yin et al. (62) added ginsenoside extract to the diet of weaned piglets, they found that the feed digestibility of weaned piglets was significantly improved compared with the control group, and the number of *Escherichia coli* in feces was significantly reduced. Ginsenoside Rb1 can inhibit porcine reproductive and respiratory syndrome virus (PRRSV) and exert its antiviral effect by interfering with RNA replication. This indicates that ginsenoside Rb1 can protect the health of sows through antiviral effects to increase the reproductive performance because PRRSV was mainly have negative effect on fertility. Kim et al. (63) found that 20 μg/mL ginsenosides Rg1 treatment improved embryo quality by culturing porcine embryo cells *in vitro*. The main mechanism is to promote the increase of glucose uptake by blastocysts through ginsenosides Rg1, and reduces the apoptosis of embryonic cells under oxidative stress conditions. This indicates that ginsenoside Rg1 can improve the survival rate of pig embryonic cells by stimulating metabolic pathways and thus indirectly improve the reproductive performance of sows. Therefore, ginsenosides can be used as a potential feed additive to improve the reproductive performance of sows.

### 4.2 The application in poultry

Adding 10, 15, and 20 mg/kg ginsenosides to the basal diet of broiler chickens aged 0–7 weeks, respectively. The results showed that ginsenosides can significantly improve the survival rate and feed utilization rate, and the best effect was achieved when 15 mg/kg ginsenosides was added (52). Another study showed that adding 300 mg/kg ginsenoside Rg1 to the diet can significantly increase the average daily gain (ADG) of yellow-feathered broilers in the late growth period and significantly reduce the feed conversion ratio (FCR) (64, 65). Adding Panax notoginseng saponins (PNS) to laying hen diets can improve egg quality. As the amount of PNS added increases, the egg white weight increases and the eggshell hardness improved. Song et al. (65) found that adding 300 mg/kg ginsenoside Rg1 to the diet could significantly increase the final body weight of broilers, reduce feed conversion rate, and improve the growth performance of broilers in the later stages. Tajudeen et al. (66) reported that adding 0.5% ginsenosides to the diet can increase the yolk content of laying hens, reduce feed conversion rate (FCR) and improve egg production performance. The reason may be that ginsenosides have a stimulating effect on oocyte meiosis and proliferation. In summary, ginsenosides can be used in poultry feed formula to improve their productive performance.

### 4.3 The application in ruminant

Studies that provide comprehensive insights into the interplay between host metabolism, gut microbiota, and feed efficiency are highly relevant to the potential applications of ginsenosides in improving livestock performance (67), especially in ruminants. There were very few literature reported the application of ginsenosides in cattle. When 1% ginsenosides were added to cattle diets, it was found that the growth performance and meat quality were improved, and there was no negative effect on other tissues or organs. The possible reason is that ginsenosides have a wide range of pharmacological activities, which can significantly improve the function of rumen fermentation, increase protein utilization and thus promote cattle growth (54). Ginsenosides Rg1 and Rg3 can inhibit bacterial reproduction by enhancing immune response and inhibiting bacterial protein signal transduction pathways, ultimately alleviating cow mastitis caused by bacterial infection (68). Ginsenosides can increase the relative abundance of beneficial bacteria in the rumen microorganisms, thereby improving the utilization of nutrients and thus increasing body weight (69). In the study on goats, intravenous injection of ginsenoside Rg1 at a dose of 1.9–2.5 mg/kg body weight into the breast can treat LPS induced mastitis. The possible mechanism is that ginsenoside Rg1 promotes binding to TLR4 and inhibits the activation of TLR4 signaling pathway by LPS, thereby exerting anti-inflammatory effects and protective effects on the mammary gland (70). Therefore, the application of ginsenosides in ruminants can enhance growth performance and also improve the quality of dairy products.

### 4.4 The application in aquaculture

**Figure 3:**
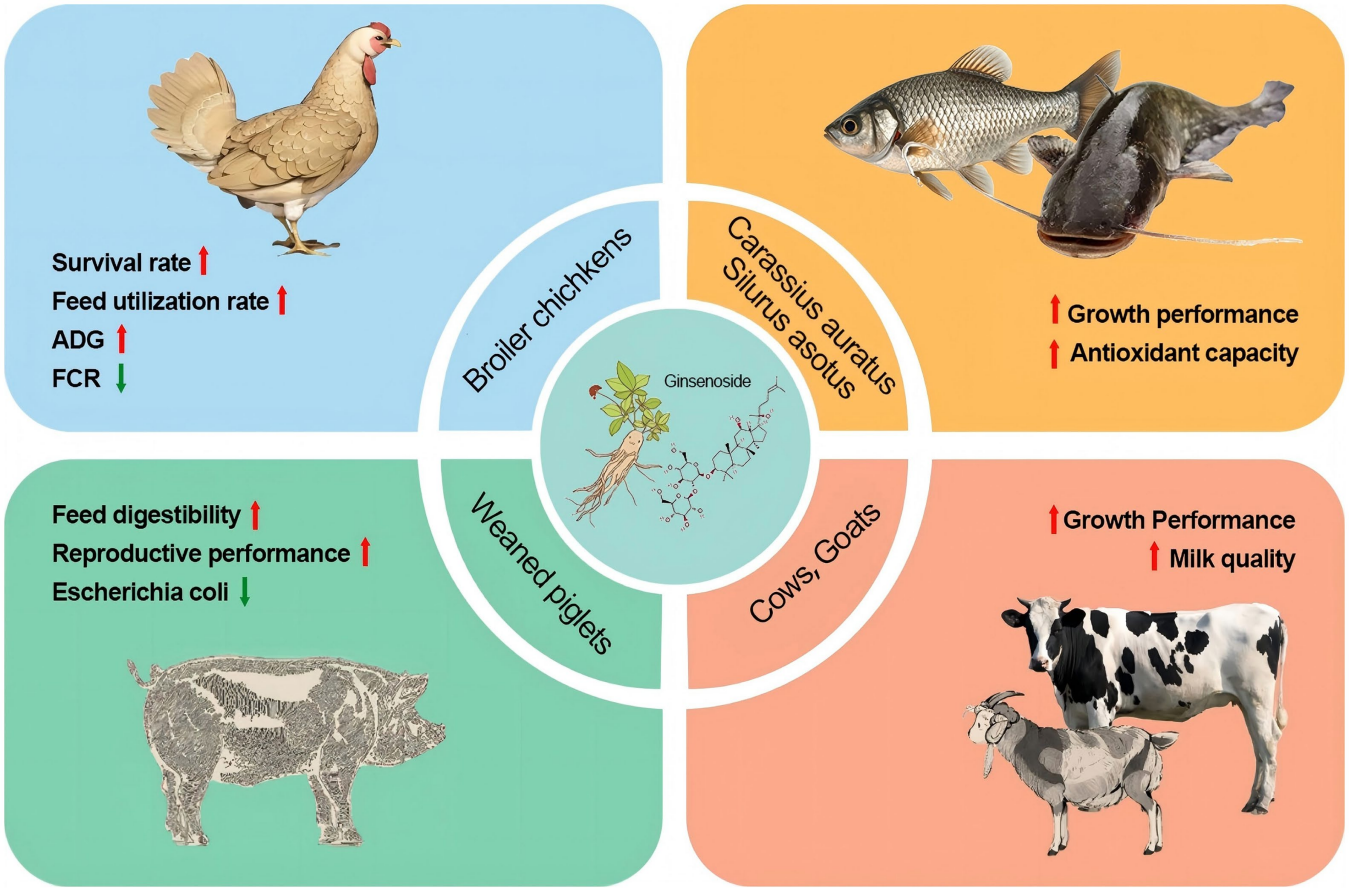
The application of ginsenosides in animal husbandry.

There are few reports on the application of ginsenosides in aquaculture. Sun et al. (71) found that the addition of ginsenosides to the diet significantly improved the growth performance and feed utilization of fish, including weight gain rate (WGR), feed efficiency ratio (FER), protein efficiency ratio (PER) and protein deposition rate (PDR). Microbial fermentation can transform ginsenosides in ginseng stems and leaves into rare saponins through deglycosylation, making them more pharmacologically active. The extract obtained by fermenting ginseng stems and leaves with *Lactobacillus casei* was added to *Carassius auratus* feed to increase the activity of GSH-Px, SOD and CAT, reduce the MDA content, and increase the gene expression levels of serum anti-inflammatory factors such as IL-10 and transforming growth factor-*β* (TGF-β) in various tissues (72). This indicated that ginsenosides can enhance the antioxidant capacity and immune-related gene expression of *Carassius auratus*. Gao et al. (73) added ginsenosides to the diet of *Silurus asotus* to explore the effect of ginseng on lipid metabolism in *Silurus asotus*. The results showed that adding 4 g/kg ginsenosides to the feed could effectively promote the growth and significantly reduce the total cholesterol and triglyceride levels in serum. The reason may be that ginsenosides can regulate the transcription level of the gene encoding iodothyronine deiodinase 2 (DIO2), reduce the synthesis of triglycerides and thyroxine, finally reduce liver fat deposition in catfish by regulating lipid metabolism (74). The above studies show that adding ginsenosides to fish diet can improve growth performance, therefore ginsenosides be used as a potential feed additive in aquaculture. The application of ginsenosides in animals was shown in Figure 3.

## 5 Conclusions and perspectives

Ginsenosides have multiple biological activities including antioxidant, anti-inflammatory, immune regulation and anti-stress. Therefore, it can be used as feed additives to improve animal growth performance and address urgent issues in the animal husbandry such as cow mastitis and piglet weaning stress. However, there are significant differences in the effects of ginsenosides on different animals, and the appropriate dosage for addition has not yet been clearly explored. The extraction process of ginsenosides is still very complicated, thus its manufacturing cost is very high. Therefore, it is very important to optimize the extraction process of ginsenosides, in order to provide theoretical support for exploring the most suitable dosage of ginsenosides in different animals and its application in aquaculture as a feed additive.
